# Can We Trust the Internet to Measure Psychotic Symptoms?

**DOI:** 10.1155/2013/457010

**Published:** 2013-07-10

**Authors:** Steffen Moritz, Niels Van Quaquebeke, Tania M. Lincoln, Ulf Köther, Christina Andreou

**Affiliations:** ^1^Department of Psychiatry and Psychotherapy, University Medical Center Hamburg-Eppendorf, Martinistraße 52, 20246 Hamburg, Germany; ^2^Department of Management and Economics, Kuehne Logistics University, 20457 Hamburg, Germany; ^3^Department of Psychology, University of Hamburg, 20146 Hamburg, Germany

## Abstract

Online studies are increasingly utilized in applied research. However, lack of external diagnostic verification in many of these investigations is seen as a threat to the reliability of the data. The present study examined the robustness of internet studies on psychosis against simulation. We compared the psychometric properties of the Community Assessment of Psychic Experiences scale (CAPE), a self-report instrument measuring psychotic symptoms, across three independent samples: (1) participants with a confirmed diagnosis of schizophrenia, (2) participants with self-reported schizophrenia who were recruited over the internet, and (3) clinical experts on schizophrenia as well as students who were asked to simulate a person with schizophrenia when completing the CAPE. The CAPE was complemented by a newly developed 4-item psychosis lie scale. Results demonstrate that experts asked to simulate schizophrenia symptoms could be distinguished from real patients: simulators overreported positive symptoms and showed elevated scores on the psychosis lie scale. The present study suggests that simulated answers in online studies on psychosis can be distinguished from authentic responses. Researchers conducting clinical online studies are advised to adopt a number of methodological precautions and to compare the psychometric properties of online studies to established clinical indices to assert the validity of their results.

## 1. Introduction

Traditionally, psychological assessments administered by an interviewer are preferred over nonpersonal assessments such as questionnaires. Face-to-face (FTF) assessment may in some cases unveil hidden symptoms and/or partly compensate for a lack of reliable information provided by a patient alone [1]. To illustrate, a patient with psychosis may deny hearing voices upon direct questioning but at the same time be observed talking to voices without overt source. This may persuade the clinician to discard the patient's response and suspect the presence of auditory hallucinations. 

On the other hand, research assessing the correspondence between self- and observer ratings indicates that self-report of psychotic symptoms is more reliable than commonly thought. Studies have generally found satisfactory associations between self- and observer-based ratings for overall pathology [2], negative symptoms [3], and positive symptoms [4–8]. 

Despite the aforementioned merits, clinical assessment is not without weaknesses. The presence of an assessor may induce important biases [9, 10], especially underreporting, that are often smaller with remote/nonpersonal measures. Further, clinicians may also underestimate depressive symptoms in acute patients with agitation or aggression or mistake primary for secondary (i.e., induced by neuroleptics) negative symptoms at times [11]. Here, self-report may be advantageous over expert ratings.

Importantly, findings obtained in clinical studies can by no means be extrapolated to the entire patient population as many patients are not willing to undergo personal assessment or treatment. In psychosis, at least one-third of individuals remain untreated [12], and patients who are nonadherent are thus likely underrepresented in conventional clinical studies [13]. Further, psychopathological and other characteristics of those who seek FTF treatment markedly differ from those who do not [14, 15]. 

To reach people with mental disorders who are unable, unwilling, or reluctant to engage in direct psychological or psychiatric contact, online assessment represents a low-threshold and economic means and may thus complement existing standard clinical assessments. However, even though studies on nonclinical samples assert that internet studies are usually as reliable as FTF contacts when certain precautions are met, many researchers still have reservations [16]. Internet studies, for instance, face the hurdle that a diagnosis is usually not externally validated [17]. Furthermore, some participants may even simulate a target disorder in order to obtain an incentive. However, the extent of simulation is considered to be low [18, 19]. After reviewing the respective literature on nonclinical studies, Hancock [16] concluded that the psychometric properties of online assessments are comparable to those of FTF interviews. Nevertheless, few data exist on the extent of manipulation in clinical (psychiatric) samples [20]. 

The present study set out to examine the reliability of data on psychotic symptoms obtained over the internet and its robustness against simulation. To meet our purpose, we contrasted the performance of three different populations (patients with validated diagnoses (face-to-face assessment), individuals with schizophrenia with a likely diagnosis (online assessment), and “simulators” (online assessment)). In line with a prior study on OCD patients [20], we expected that results obtained from an online sample of patients with a likely diagnosis of schizophrenia would be equivalent to those obtained from a sample with validated diagnoses thereby confirming the reliability of online assessment. In contrast, simulators were expected to overreport symptoms and to show inflated scores on a newly designed lie scale [9]. 

## 2. Methods

### 2.1. Participants

#### 2.1.1. Patients with Schizophrenia with Verified Diagnoses (Sample 1)

In the framework of a study on the cognitive effects of stress induction, we recruited a sample of individuals with schizophrenia spectrum disorders (*n* = 33) whose diagnoses were verified using the Mini Neuropsychiatric Interview (MINI; Sheehan et al., [21]), complemented by chart review. All participants were inpatients treated for schizophrenia in hospitals from the Hamburg and Marburg Metropolitan areas (Germany). Patients were between 18 and 65 years old, able to provide informed consent, had good command of the German language, IQ > 85, displayed no diagnosis of bipolar disorder and substance dependence (last six months; diagnostic information was verified with the MINI interview), and showed no macroscopic neurological disorder (14 females, 19 males; age: M = 40.42 years (SD = 12.17)). All of these participants were interviewed with the Positive and Negative Syndrome Scale (PANSS) [22] and asked to complete the CAPE (see below) at baseline, that is, before the experimental phase began. The CAPE was administered in its original paper-and-pencil format.

#### 2.1.2. Individuals with Schizophrenia with Probable Diagnoses (Sample 2)

Data for the second sample was derived from a recently published internet study on medication adherence (for details, see [9]). Participants were recruited online via posts on several moderated German online discussion forums providing people with psychosis with the opportunity to exchange information online. A web link provided access to the internet questionnaire. Participation was strictly anonymous to foster unbiased responses. When accessing the internet survey, participants were first asked for background information (e.g., age) as well as their medical history (e.g., medication). “Cookies” prevented multiple accesses from the same computer. Participants who failed to complete the questionnaire, had no diagnosis of schizophrenia (self-report), or admitted that they had not answered openly were excluded. Data from 113 participants were considered for the final analyses (females 39, males 74; age: M = 37.15 (SD = 9.55)). Data of three participants who achieved more than 8 out of 16 points on the four lie scale items (see below) were deleted (this will be considered in the analyses on the lie scale). 

#### 2.1.3. Simulators (Sample 3)

We recruited a sample of distinguished experts or students who were asked to simulate a diagnosis of either OCD (not relevant for the present study; this part of the investigation has been published in [20]) or psychosis. Via email, the first author contacted medical and psychology students from the University of Hamburg (Germany) who had attended a curriculum on mental disorders including psychosis. In addition, we contacted distinguished experts (i.e., persons who had actively engaged in research on schizophrenia, worked with patients with schizophrenia, and/or had written original research articles on schizophrenia). The invitation was emailed to specific individuals in order to ensure that only persons with some expertise would take part in the survey. These persons were asked to complete questionnaires via the internet. The survey was constructed using the software package Unipark. Two scales on OCD [20] were followed by a scale on the (subclinical) psychosis phenotype (CAPE; see next). Participation was strictly anonymous to foster unbiased responses. Participants were instructed to answer the CAPE items as if they had schizophrenia. There were no constraints on whether participants should simulate a patient with acute or remitted symptoms or with respect to the specific clinical picture. At the beginning of the assessment, participants were also asked about their knowledge about schizophrenia and their source of expertise. To increase the probability of successful simulation (i.e., to make the design more conservative), we disclosed the purpose of the study (i.e., whether or not it is possible to simulate a clinical disorder) to participants beforehand. Informed consent was obtained online from all participants in accordance with the requirements of the local department of data security and the local ethics committee in Hamburg (Germany).

#### 2.1.4. Community Assessment of Psychic Experiences Scale (CAPE)

The Community Assessment of Psychic Experiences Scale (CAPE; [23]) consists of 42 items (four-point Likert scale: “Never,” “Sometimes,” “Often,” and “Nearly always”) that tap into the psychosis phenotype [24]. The CAPE has three subscales that measure psychotic (item 5 “Do you ever feel as if things in magazines or on TV were written especially for you?”), negative (item 8: “Do you ever feel that you experience few or no emotions at important events?”), and depressive (item 9: “Do you ever feel pessimistic about everything?”) syndromes. The reliability and (factorial) validity of the scale are good [25, 26]. We used the authorized German translation of the CAPE. The translation corresponded to the original instruments with respect to instruction and content. We added four lie scale items [9] mirroring common misconceptions about psychosis (cutoff: 8 points): (a) seeing tiny objects like white mice (indicating delirium rather than psychosis); (b) alien abduction (a rare but highly publicized (face-valid) cliché symptom), (c) being a famous historical personality (a rare but highly publicized (face-valid) cliché symptom), and (d) mental lapses during which one becomes another person (i.e., split personality; a rare/implausible but highly publicized cliché symptom). Scores beyond the cutoff speak for simulation of psychosis and/or unreliable responses.

## 3. Results

Table 1 shows the psychometric indices of the CAPE across the different samples. All samples achieved good scores with respect to internal consistency. Moreover, simulators largely exceeded the clinical samples on positive syndrome scores and on the lie scale (available for Samples 2 and 3 only). The proportion of (probable) imposters was at least four times higher in the simulator group compared to the patients groups (see Table 1). The intercorrelation for negative and depression scores was high in all groups. In contrast, zero correlations emerged between the positive syndrome with both the depressed or negative syndrome in the simulator group, whereas these syndromes were significantly related in the patient samples. 

## 4. Discussion

With the aid of statistical procedures, scientific studies aim to generalize from a (usually small) sample to the entire population. As outlined in the introduction, results from neither clinical nor internet studies targeting mental disorders can claim full representativeness as, for example, patients seeking help (those usually examined in clinical studies) and those who do not (those more easily recruited via internet studies) seem to differ on important characteristics [14, 15]. Still, there is reluctance to consider internet studies as a complementary methodological tool. 

Therefore, the present study set out to test the robustness of internet-based research in schizophrenia against simulation. As expected, distinguished experts had a marked tendency to overreport positive (but not negative and depressed) symptoms and, unlike clinical samples, the positive syndrome did not correlate with negative and depressed symptoms (This seems to contradict results from factor analysis suggesting independent dimensions. However, orthogonality is a mathematical constraint when using varimax rotation, a common method in factor analysis. Still, raw data usually show that different syndromes are intercorrelated: higher symptom severity in one psychopathological domain is accompanied by higher symptom severity in other domains as well). The mean scores of the schizophrenia samples were clearly above the values reported for nonclinical controls (e.g., [26]; (please note that Fonseca-Pedrero et al. (2012) report sum values which have to be divided by the number of items to allow comparison with the present sample)) and in the range of previously reported CAPE scores in clinical samples (e.g., [26, 27]) confirming the validity of the results. Finally, our newly devised psychosis lie scale capturing pseudopsychotic symptoms distinguished real patients from simulators. To conclude, psychometric characteristics of the patient samples can be considered as good, confirming earlier claims that the responses of patients with schizophrenia in online assessments are more reliable than commonly thought and that self-report assessments represent an important source of information [8, 28–30].

A number of limitations need to be acknowledged. First, our study was not designed to identify specific individuals who feign psychotic symptoms. Second, the present findings cannot be fully extrapolated to scales other than the CAPE. Third, patient Sample 1 was rather small and was not assessed with the psychosis lie scale. 

While our study suggests that internet studies are better than their reputation and represent an important complementary approach to conventional research, we still recommend several measures to decrease the risk of simulation and to detect potential simulators (“imposters”) in such studies. For example, the incorporation of lie scales as well as plausibility checks (e.g., requesting the same information twice in disguised form, for example, age and date of birth) may help to filter out simulators. Moreover, subjects who enter the same value for all items should be excluded. Overall, the consequences of false-positive judgments (i.e., inclusion despite invalid data) are deemed more grave than false-negative assignments in internet studies. A nonmonetary compensation for participation (e.g., self-help manual for the respective disorder) may also ward off simulators who are solely interested in financial reimbursement. Moreover, patients are best recruited from specific sources (e.g., online forums for schizophrenia rather than general forums for mental disorders). 

As shown, several psychometric indices may serve as a proxy to determine the reliability of internet samples. If the psychometric properties of an internet sample are similar to established scores, this speaks for the validity of the results. Where possible, additional telephone interviews should be considered, which however raises the threshold of recruitment considerably. Another option is to ask in- and outpatients with a verified diagnosis for their email address before discharge and whether they are willing to participate in internet studies or to ask subjects to send blinded diagnostic information via fax or email [31]. 

## Figures and Tables

**Table 1:** Psychometric properties (in square brackets: results for patient groups [Samples 1 and 2] versus “simulators” [Samples 3a and 3b, see text for explanation]).

Variables	Sample 1 (verified Sz diagnosis; *N* = 33)	Sample 2 (probable but unverified Sz diagnosis; Moritz et al., in press, *N* = 113)	Sample 3a (full sample, simulators, *N* = 121)	Subgroup 3b (distinguished experts only, *n* = 86)	Statistics; post-hoc Bonferroni corrected
*Means (weighted score; standard deviations) *					
CAPE positive (range 1–4)	1.86 (0.49)	1.72 (0.46)	2.57 (0.60)	2.59 (0.62)	*F*(3,263) = 54.66, *P* < .001; [Samples 3a/b] > Samples 1 and 2
CAPE negative (range 1–4)	2.26 (0.59)	2.28 (0.53)	2.24 (0.54)	2.26 (0.54)	*F*(3,263) = 0.13, *P* > .9
CAPE depression (range 1–4)	2.38 (0.63)	2.28 (0.50)	2.33 (0.49)	2.33 (0.50)	*F*(3,263) = 0.44, *P* > .7
CAPE lie scale (range 1–4)	n.a.	1.24 (0.27) [5.3% ≥ 8, 2.6% ≥ 9]*	1.59 (0.63) [23.1% ≥ 8, 15.7% ≥ 9]	1.59 (0.66) [20.9% ≥ 8, 17.4% ≥ 9]	*F*(2,231) = 14.43, *P* < .001; [Samples 3a/b] > Sample 2 (at least *P*< .05)

*Internal consistency (Cronbach's α*)					*Correlational differences *
CAPE positive	.89	.89	.93	.94	*P* > .1
CAPE negative	.90	.89	.90	.89	*P* > .1
CAPE depression	.88	.80	.79	.79	Sample 1 > [Samples 2 and 3a/b] (at least *P*< .05)

*Intercorrelations CAPE subscales *					*Correlational differences *
Positive-negative	.61 (*P* < .001)	.35 (*P* < .001)	−.08 (*P* > .3)	−.09 (*P* > .4)	[Samples 1 and 2] > [Samples 3a/b] (at least *P*< .05)
Positive-depressed	.71 (*P* < .001)	.43 (*P* < .001)	.04 (*P* > .6)	.03 (*P* > .7)	[Samples 1 and 2] > [Samples 3a/b] (at least *P*< .05)
Negative-depressed	.78 (*P* < .001)	.62 (*P* < .001)	.69 (*P* < .001)	.67 (*P* < .001)	*P* > .1

Notes. CAPE: Community Assessment of Psychic Experiences.

*Three subjects with scores of 8 were removed from the final sample.
